# Twice-daily Radiation for Metastatic Malignant Melanoma: A Different Approach Resulting in a Significant Response

**DOI:** 10.7759/cureus.4161

**Published:** 2019-03-01

**Authors:** Norman H Anderson, Julie B Arcaro

**Affiliations:** 1 Radiation Oncology, Robert Boissoneault Oncology Institute, Ocala, USA

**Keywords:** malignant melanoma, metastatic malignant melanoma, radiation therapy, hyperfractionation, twice-daily radiation, abscopal effect

## Abstract

The aim of this study was to discern an abscopal effect by modifying the delivery of radiation for metastatic malignant melanoma. The effect would be directly evident with visible/radiographic regression of the disease and indirectly shown with an overall extension in survival and potential cure. Patients with locally advanced, metastatic palpable, or radiographic visible metastatic malignant melanoma were treated with twice-daily radiation therapy using a dose range of 100-135 centigray (cGy) per fraction. A 100% complete response/continued regression with no recurrence was achieved within the region of delivery for every patient so treated. Of those alive at three years, few demonstrate a progression of the disease. These results were achieved without the use of immunotherapy, created few side effects, and were accomplished at a fraction of the alternative’s cost. Evidence of an immune-mediated response (abscopal effect) was commonly seen. Treatment was administered within acceptable dose ranges, historically used twice daily for other malignancies known to be sensitive to the effects of radiation.

## Introduction

The concept of using radiation twice-daily for head and neck carcinomas began at the University of Florida. Such cancers are known to be sensitive to once-daily radiation. It was believed that if the amount of energy was reduced for each individual treatment, and delivery occurred twice-daily rather than once, such an approach may allow an increase in the total amount of energy safely administered, resulting in a better cure. Decades of research confirmed that for squamous cell malignancies of the head and neck, this was indeed the case [1]. At the same time, less long-term side effects occurred to uninvolved but exposed normal tissue, demonstrating the safety of such an approach.

For almost half a century, malignant melanoma, in contrast, has proven resistant to radiation. When standard amounts of radiation known to destroy head and neck, breast, and lung cancers are administered once-daily for malignant melanoma, it doesn’t work. Administering reduced or “weaker doses” twice-daily to improve results would defy logic.

In essence, the direction evolving over time seeks to administer radiation by increasing the amount given with each once-daily dose but delivering the total over very few treatments [2-7]. Forty years of this approach for malignant melanoma, referred to as hypofractionation, provides a slight improvement in disease control. The role of radiation has historically been applied for the risk of residual microscopic disease following surgical resection. Historically, the most commonly used combinations are chemotherapy/radiation. The experience with targeted therapy and immunotherapy is still more limited as is the recent use of immune and/or targeted drugs combined with hypofractionation. However, damage to the normal tissue with this combination can be significant, and in light of radiation’s minimal benefit in this setting, the resultant side effects may limit its application.

Over 59,000 people worldwide die each year from metastatic disease [8]. Here, we set out to test the hypothesis that in a group of patients with unacceptable or no treatment alternatives, could a different delivery of radiation by itself change results?

The current study was inspired over two decades ago by an anecdotal case of a 38-year-old male who presented with 23 separate brain lesions. Representative biopsies confirmed metastatic malignant melanoma, consistent with his original skin lesion of similar pathology. Realizing once-daily radiation for this cancer to be ineffective and due to the diffuse nature of parenchymal involvement, low-dose twice-daily treatment of the entire brain was administered. Follow-up radiographic scans revealed all lesions to have completely regressed in a patient now without symptoms or signs of neurologic sequelae. He was lost to follow-up more than seven years thereafter, without evidence of disease. Unfortunately, records are no longer available and thus he is not included in the evaluation, but the unique and unexpected response led to the design of the study and justification for enrollment of the presently included patients. As cases referred by other physicians or as patients themselves presented for consideration and subsequent treatment, extended survival with reduced occurrence of any malignancy became evident in a diagnosis whose very nature is that of progressive spread. Close physical and radiographic examination revealed complete tumor response in the area of prior treatment, a reduction in further evidence of melanoma elsewhere, a lack of systemic side effects, and increased overall survival. The abscopal effect is a historically rare occurrence and is defined as the resolution of an untreated similar malignancy resulting from definitive radiation treatment administered elsewhere. It was originally described by RH Mole in 1953 [9]. This phenomenon, where radiation and immunotherapy are combined, has recently gained interest. Studies on this are presently ongoing.

The evaluation of the included study patients now supports the hypothesis for this effect when treating a metastatic malignant melanoma, that is, twice-daily radiation treatment alone delivered within defined parameters uniquely precipitates an immune response termed the abscopal effect, which may be the singular basis for complete tumor regression, reduced occurrence of disease elsewhere, elimination of untreated but known disease, increased survival, and potential cure.

## Materials and methods

Design of individualized treatment

Historically, patients have received treatment twice-daily. As mentioned previously, this application for head and neck malignancies is well-founded. Similarly, this approach has been used for lung carcinoma as well as glioblastoma. The delivery of twice-daily radiation has been used for malignant melanoma, but at doses per fraction much greater than used for these select patients. In addition, the tolerance of normal structures requires decreased doses when administered more frequently than once a day. Those doses administered for the included patients are within the established standard of care.

Treatment of these study patients was not a protocol. Rather, those receiving intervention initially presented by referral from a physician who sought some form of treatment for the patient when no other alternatives were available or the patient themselves sought evaluation for possible treatment.

Nineteen patients are included in this study. They evidenced gross visible and/or palpable disease, demonstrated radiographic evidence of metastatic disease confirmed by biopsy to be malignant melanoma, and refused systemic intervention or were not medically eligible. Since this was not a protocol, there were no exclusion criteria, as all patients who presented with metastatic disease regardless of site or advanced stage were treated and analyzed within the results. The first patient in the study was treated in mid-August to early October 2007, with the last enrolled in October 2018. Twenty-one patients treated for microscopic or known sub-clinical residual disease in the post-operative setting are not included in this evaluation, but all attained complete control.

Each patient was informed that their care would be individualized since treatment was not administered as a protocol. The principles of radiobiology were explained in detail, using understandable analogies relevant for each patient. The concept of once-daily fractionation was reviewed. The difference with twice-daily administration was then illustrated, emphasizing both total dose as well as reduced long-term adverse effect to normal tissue when less radiation was administered with each fraction. It was explained that identical doses had been used for other malignancies but had not been applied to malignant melanoma. Thus, although doses were administered twice-daily within the standard of care, the application for malignant melanoma as specific cancer had not been used since the standard once-daily dosage was historically ineffective. However, the tolerance of normal tissue would remain independent of the malignancy treated. Patients were treated twice-daily using Varian linear accelerators (Varian Medical Systems, Palo Alto, California, USA) and included photon and/or electron energy based upon the region of anatomy requiring treatment. The design of each individual patient's treatment remained identical, whether administered once versus twice daily and conformed to existing standards of care.

## Results

This is a retrospective study of patients who were treated twice-daily using photon, electron, or a combination of energies. Since some patients received more than one area of treatment, sites receiving photons versus electrons varied. As all 19 patients in this study’s analysis demonstrate at a minimum complete disappearance of localized disease and with survival, the first three highlighted patients in order serve to most clearly document key components. These include complete/sustained resolution of significant visible malignancy with long-term survival and no evidence of further disease; initial elevated metabolic activity/increased anatomic size consistent with lymphatic spread beyond the treatment volume which, on follow-up pathologic review, demonstrates an immune inflammatory response and complete absence of malignancy; and documented metabolic activity/anatomic mass effect within the lung, with subsequent complete/sustained radiographic resolution without ever receiving direct treatment. The final fourth patient demonstrates complete local response suggestive of an abscopal effect in the lung, but the trend for metastases to occur in the abdomen.

Patient one

**Figure 1:**
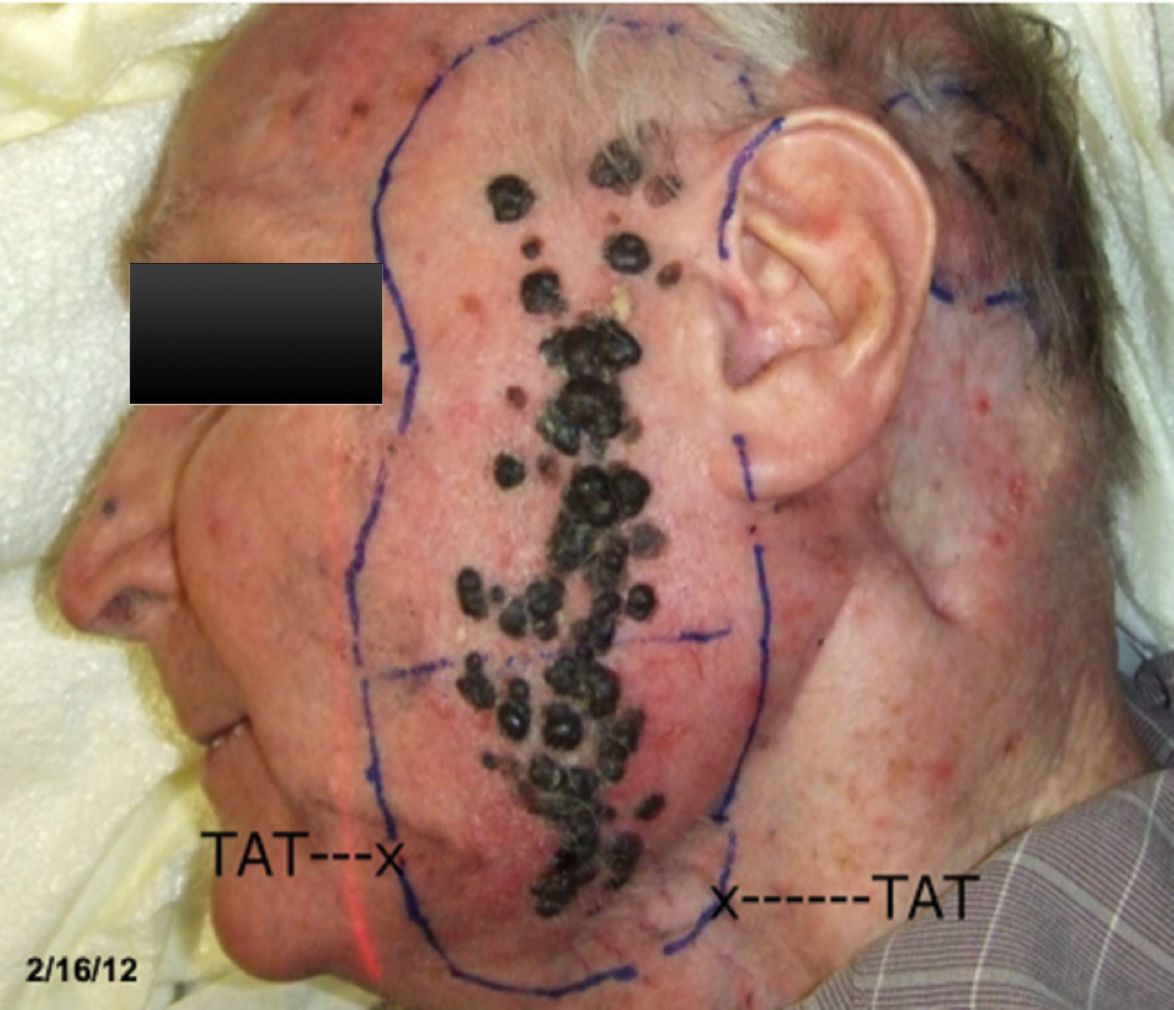
Prior to initiation of treatment

In February of 2010, an 81-year-old gentleman presented with melanomas of the left post-auricular region and left scalp. Radical surgery with full thickness grafting was performed. Remaining disease required an immediate second operation, with further surgery in August. Recurrences in March through September of 2011 required multiple additional surgeries, performed at a leading academic center. Molecular testing revealed a BRAF V600E mutation. Thereafter, the patient was begun on vemurafenib, but experienced significant side effects, as the cancer's visible growth progressed. All treatment was discontinued in December of 2011.  First seen by the authors in February of 2012 (Figure 1), the patient subsequently received twice-daily 6 Mev electron therapy. The fields were templated to both the left face and left posterior scalp, conformed with lead shielding, and treated appositionally through 1 cm of bolus from February through March of 2012, for a total dose of 5940 centigray (cGy), at 135 cGy/fraction. No further pharmaceuticals during or following radiation were given nor further surgery performed.

**Figure 2:**
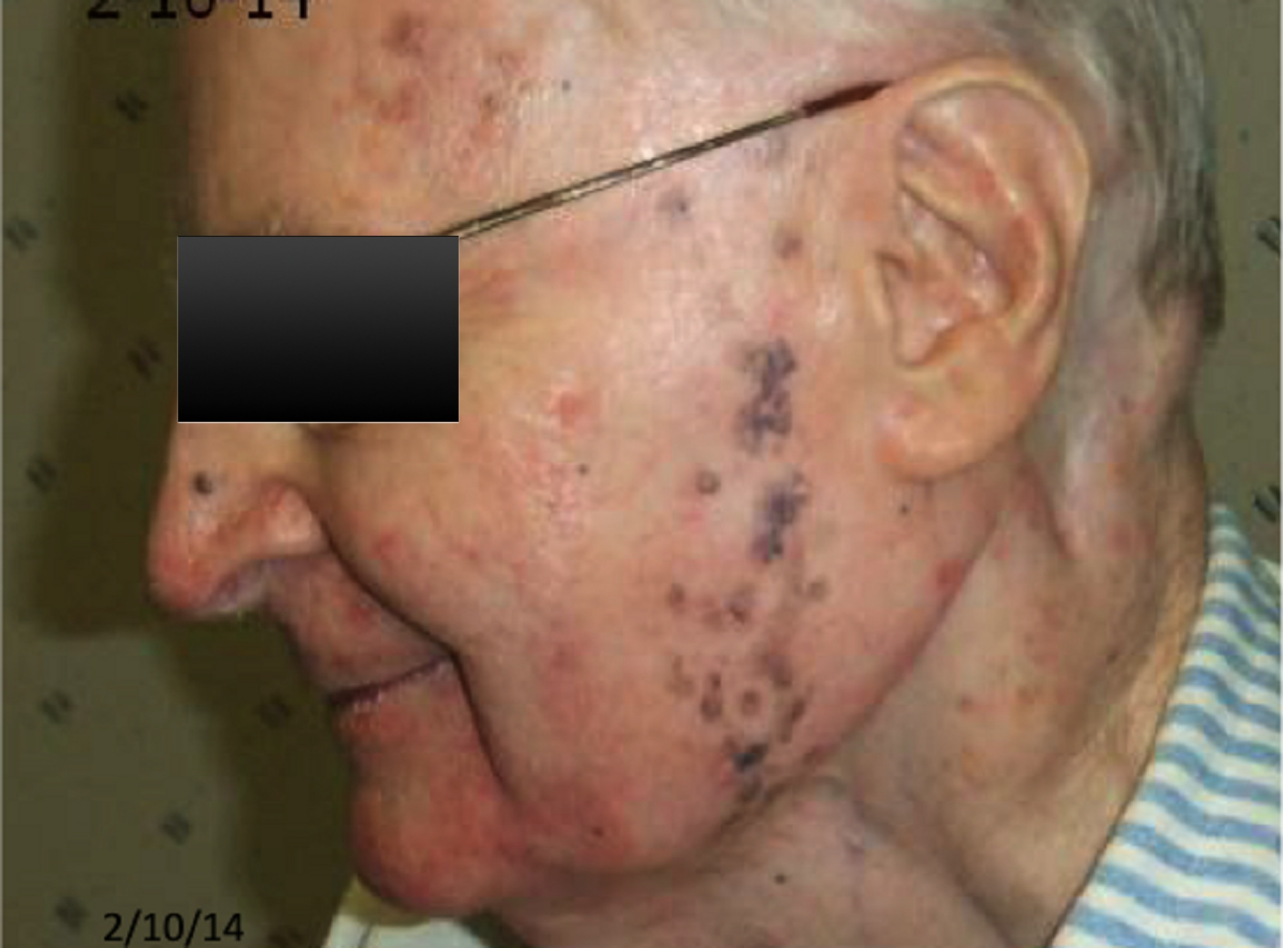
Two years post-treatment

**Figure 3:**
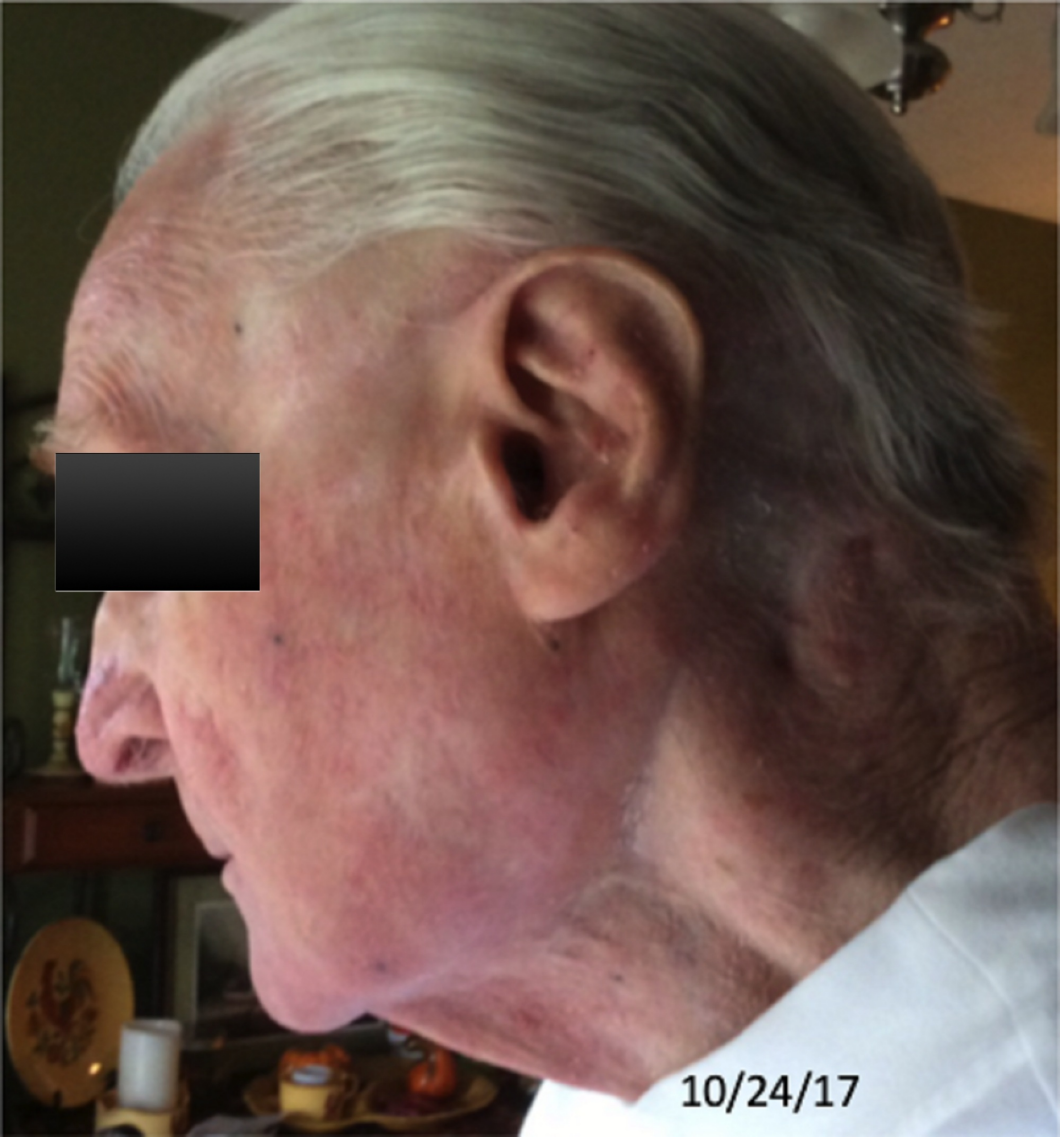
Six years post-treatment

Although the patient did not have further evidence of metastatic disease at the time of treatment, physical exams, as illustrated in Figures 2-3, and positron emission tomography-computed tomography (PET/CT) scans find our patient to be without evidence of recurrence or metastatic disease: increased survival in a patient who seven years earlier had no treatment alternatives. It may be an assumption that lack of further disease indicates an immune response. But with this patient's number of recurrences, and staged as IIIc metastatic disease prior to treatment, these results are indicative of such a response. In addition, there has been excellent maintenance of the skin with no scar tissue formation. There has been no deficiency in the patient's taste, smell, hearing, or ability to swallow.

Patient two

A 59-year-old female was diagnosed with a melanoma on her face, with the lesion resected in 1999. In June of 2007, episodes of visible blood in the urine occurred. The patient described a feeling of fullness in the pelvis. Her vaginal exam revealed a very large, fungating mass attached to the bladder. The biopsy was reviewed at Mayo Clinic, Rochester and confirmed melanoma. Blood in the urine continued, as vaginal bleeding increased to an average of four to five pads daily. Of note, the patient underwent a total abdominal hysterectomy with partial removal of her ovaries many years before her diagnosis of malignant melanoma.

**Figure 4:**
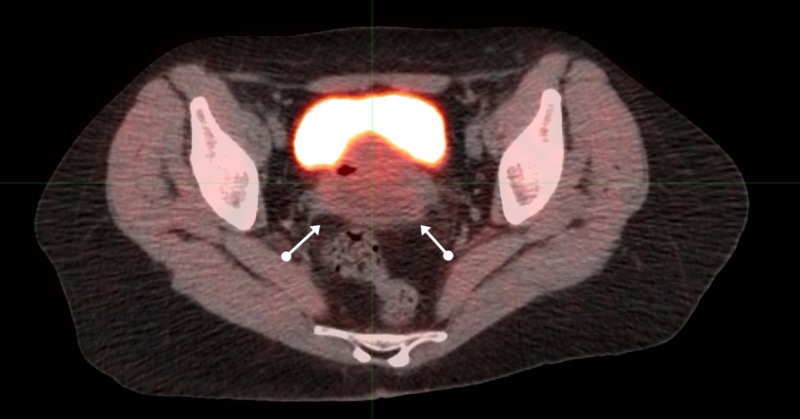
Sequential axial PET/CT images of pelvic malignancy: proximal positron emission tomography-computed tomography: PET/CT

**Figure 5:**
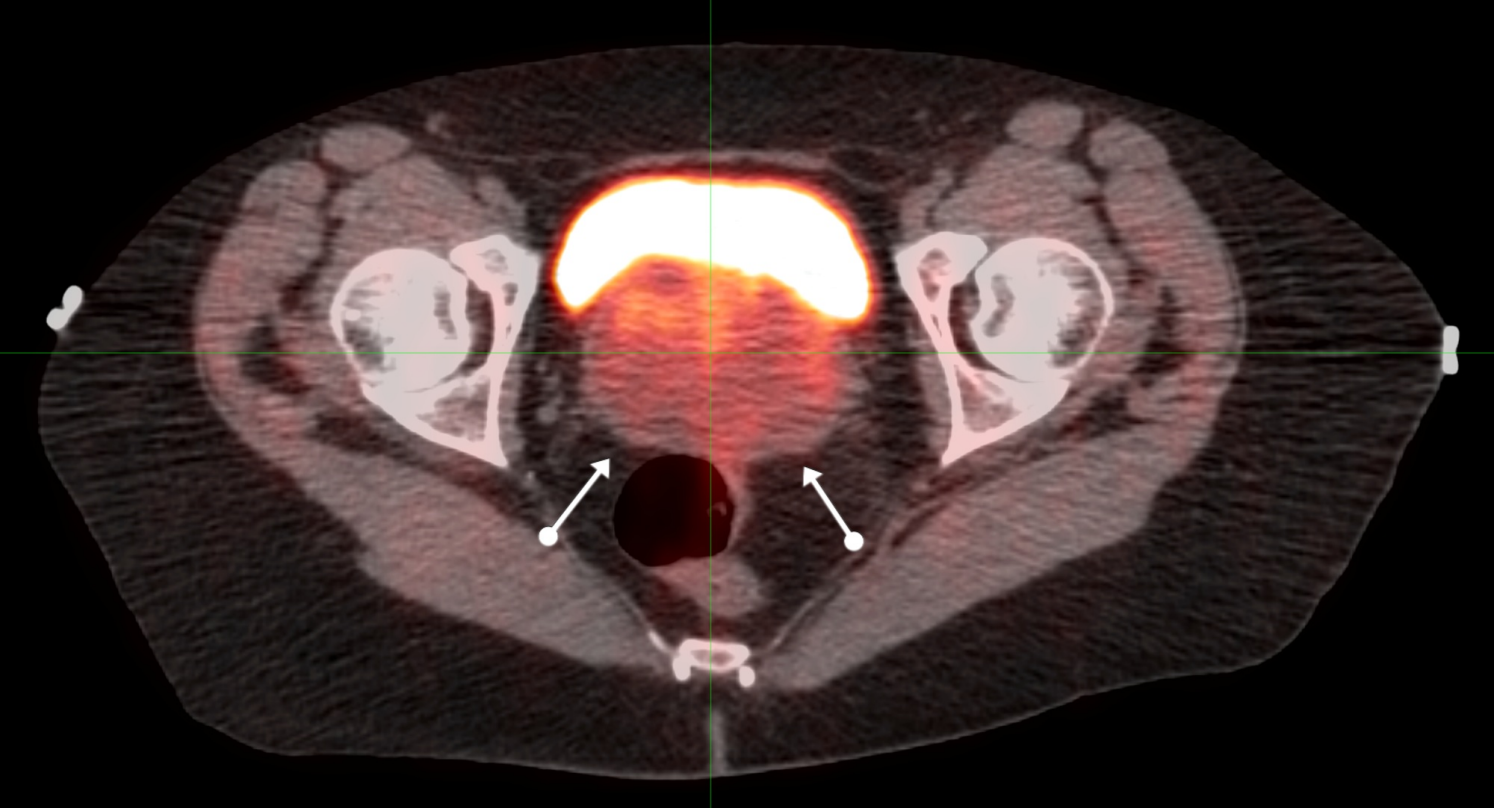
Sequential axial PET/CT images of pelvic malignancy: mid positron emission tomography-computed tomography: PET/CT

**Figure 6:**
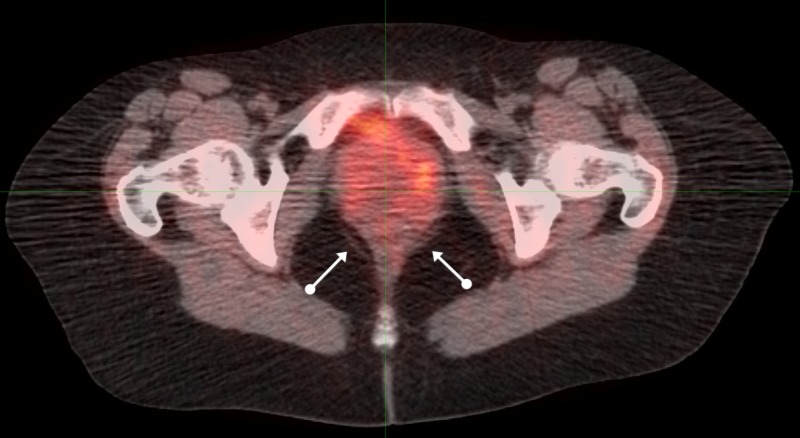
Sequential axial PET/CT images of pelvic malignancy: distal positron emission tomography-computed tomography: PET/CT

Computed tomography (CT) and magnetic resonance imaging (MRI) of the pelvis in July of 2007 revealed a 7 x 6 cm pelvic mass connected to the anterior wall of the rectum, inseparable from the posterior wall of the vagina. The PET/CT of August 3, 2007, (Figures 4-6) revealed a similar pelvic mass with elevated activity of 5.9 units.

Twice-daily radiation to a total dose of 7440 cGy was delivered twice-daily at 120 cGy for 42 fractions to 5040 cGy: 100 cGy twice-daily was then delivered to a reduced field to 2400 cGy: all treatment was administered from August through October of 2007.

**Figure 7:**
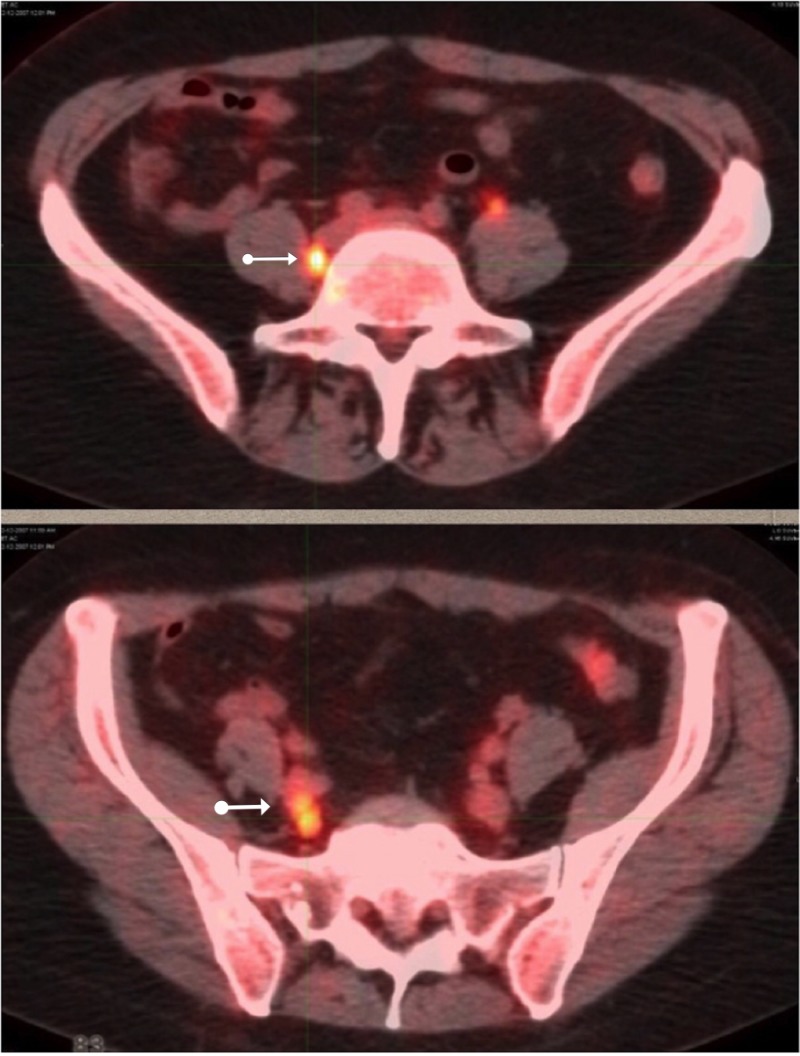
PET/CT three months post-treatment positron emission tomography-computed tomography: PET/CT

A post-treatment PET/CT in December (Figure 7) revealed several new lymph nodes along the right psoas muscle and right common iliac vessels, with metabolic activity of five units; elevated levels not consistent with just inflammation. These lymph nodes had not been included in the original radiation treatment volume since they were beyond the pelvis. The patient underwent surgical removal. None of the metabolically active lymph nodes to include the right common, right aortic, left periaortic, left common, or right external iliac nodes contained any evidence of malignancy, indicative of but not conclusive for immune-generated tumor destruction.

No chemotherapy or immunotherapy was ever administered. The absence of any further malignancy now, 11 years later, reflects an inflammatory reaction at the minimum and is indicative of an abscopal effect.

Patient three

A 72-year-old male dentist underwent resection of malignant melanoma from the left eyebrow in 1997. In March of 2013, resection of a second melanoma was performed and it was felt to be a new metachronous lesion. Six re-resections were required with the last in September of 2013. In April of 2014, a recurrent left facial metastatic node lateral to the left orbit was excised. On May 20, 2014, a left parotidectomy and left neck dissection, including levels IV and V, were completed, with pathology negative. Follow-up PET/CT in February of 2015 was negative. In August, a PET/CT revealed a 9 mm right upper lobe pulmonary nodule, with wedge resection confirming malignant melanoma. From November of 2015 to January of 2016, ipilimumab, an immunotherapeutic agent, was administered. But less than one month later, a PET/CT revealed two new contiguous left neck Level V hypermetabolic metastatic lymph nodes. Biopsy confirmed malignant melanoma.

**Figure 8:**
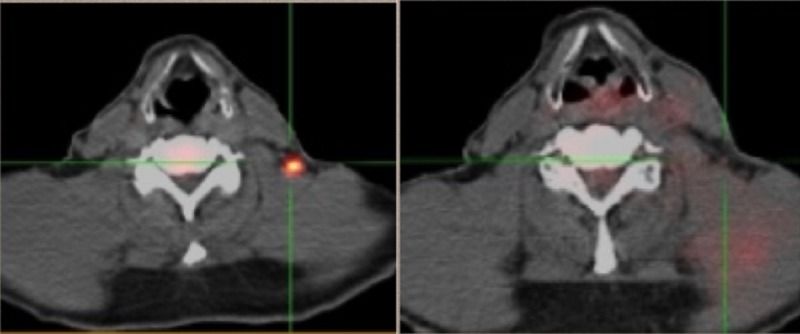
Pre/post PET/CT positron emission tomography-computed tomography: PET/CT

From March of 2016 to April of 2016, BID radiation to the left neck Level V lymph nodes was delivered. In May, CT findings showed resolution (Figure 8). But now two new left lung nodules were evident. No treatment was administered.

**Figure 9:**
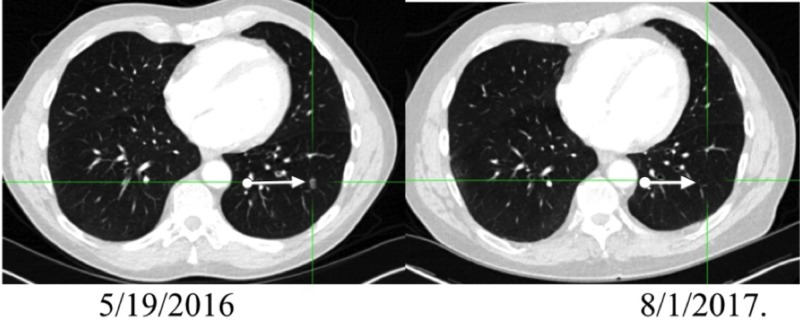
Resolution of left lower lobe pulmonary nodule

By July, a repeat PET/CT scan was negative, as have all radiographic studies since, indicative of an abscopal effect (Figure 9), although the reactions in the lung could have been reactive. Since he did have prior metastatic malignant melanoma in his lung resected, it is logical to assume that further involvement may have occurred, and thus the high index of suspicion. This was a patient who recurred every few months, now approaching 3 years without further malignancy evident.

Of patients succumbing to this malignancy, the last selected example documents complete local response in each area treated, but decreased survival correlating with metastases to the abdomen.

Patient four

In June of 2015, an 83-year-old retired female nurse noticed an ulcerative lesion involving the skin of her right ankle. Biopsy revealed Clark level IV melanoma with a thickness of 1.8 mm.

**Figure 10:**
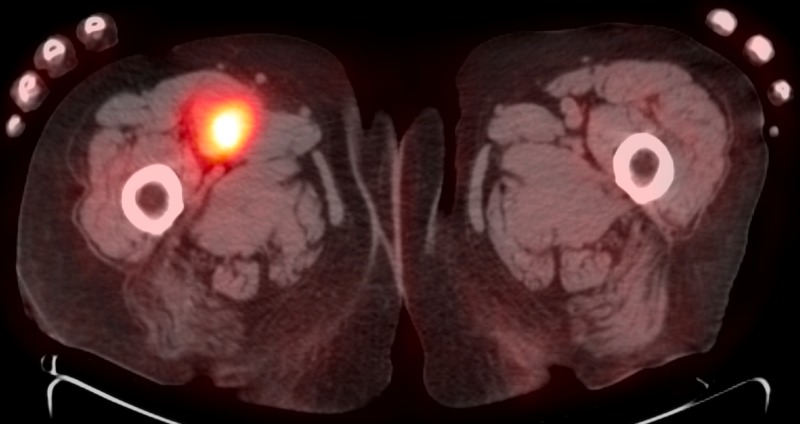
PET/CT of August 25, 2015 positron emission tomography-computed tomography: PET/CT

The original PET/CT of August 2015 demonstrated soft tissue thickening with subcutaneous edema. Numerous metabolically active soft tissue nodules in the right lower extremity extended to the proximal femoral artery (Figure 10). Performed at the University of Florida, this study documented "a bulky soft tissue mass in the proximal anterior compartment of the right thigh measuring 4.7 x 4.0 cm, encasing the femoral artery, and revealing an SUV of 7.3 units,” as she was diagnosed with stage IIIc disease. A leading academic institution recommended a radical lymphadenectomy of the right lower extremity, extending into the groin. The patient refused surgical intervention. Ipilimumab could not be used due to the patient's compromised renal status.

**Figure 11:**
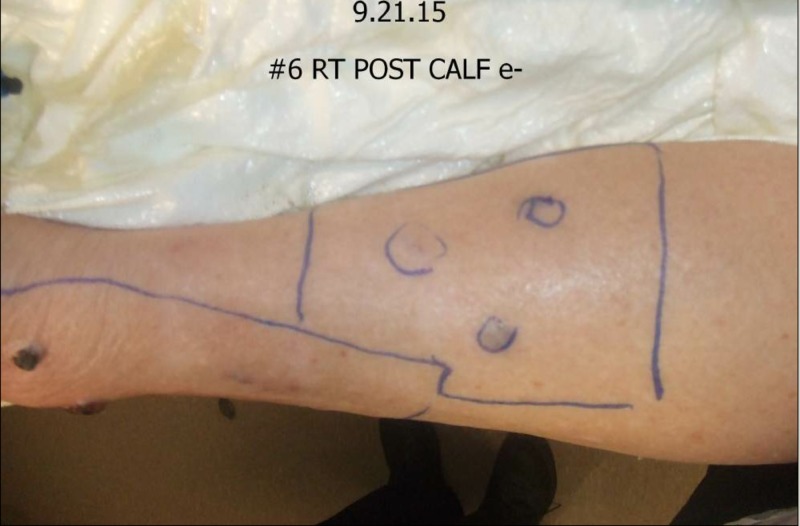
Physical exam September 21, 2015

**Figure 12:**
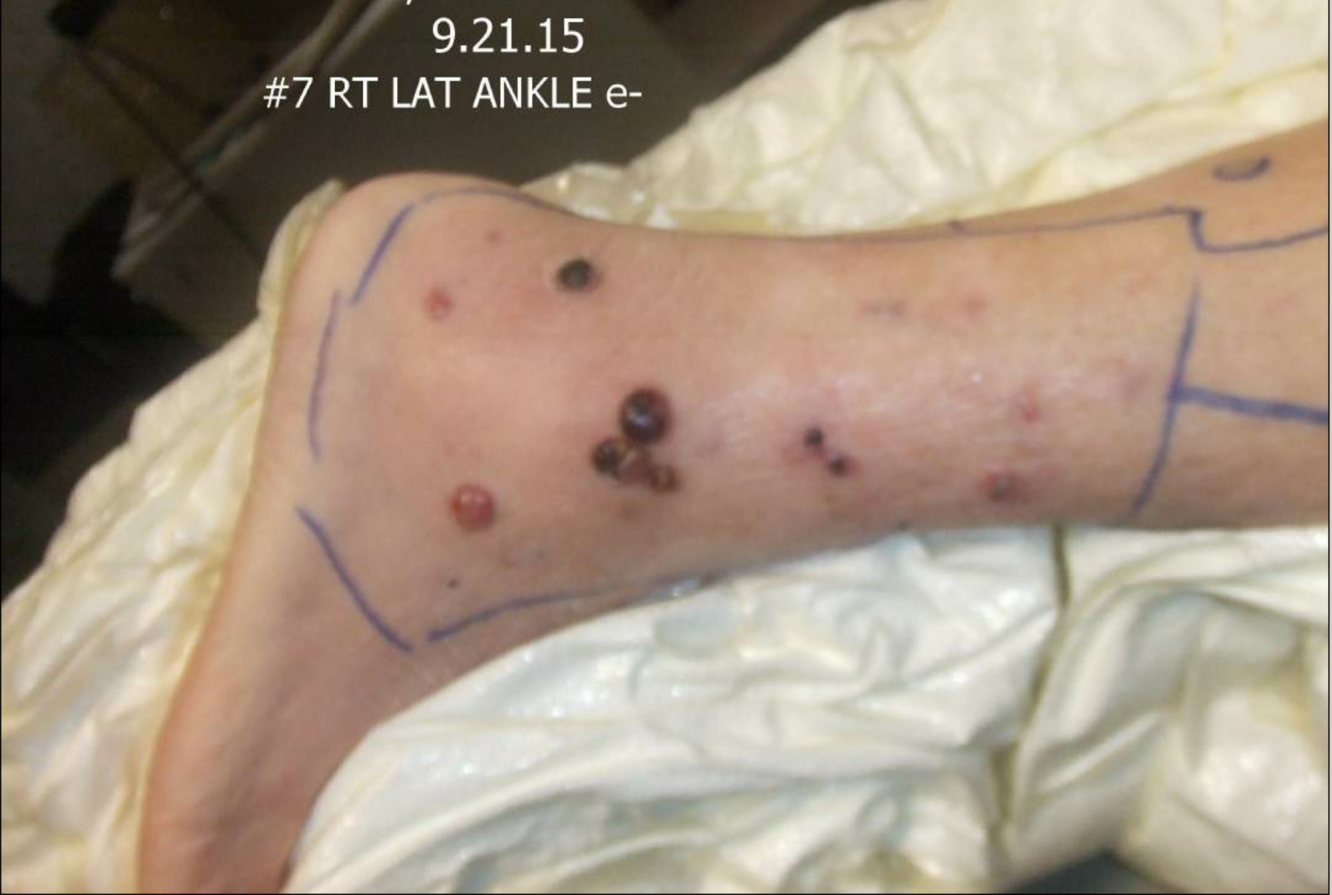
Physical exam September 21, 2015

First seen in September of 2015, her physical exam revealed elephantiasis of the right lower extremity. Multiple cutaneous and subcutaneous nodules could be palpated to the level of the groin, with individual adenopathy greater than 4.0 cm along the medial aspect of the right thigh (Figures 11-12).

She received twice-daily radiation to regions of the right lower extremity and pelvis to include visible, palpable, and radiographic disease with total doses ranging from 3645 cGy to 5265 cGy at 120 cGy to 135 cGy per fraction.

**Figure 13:**
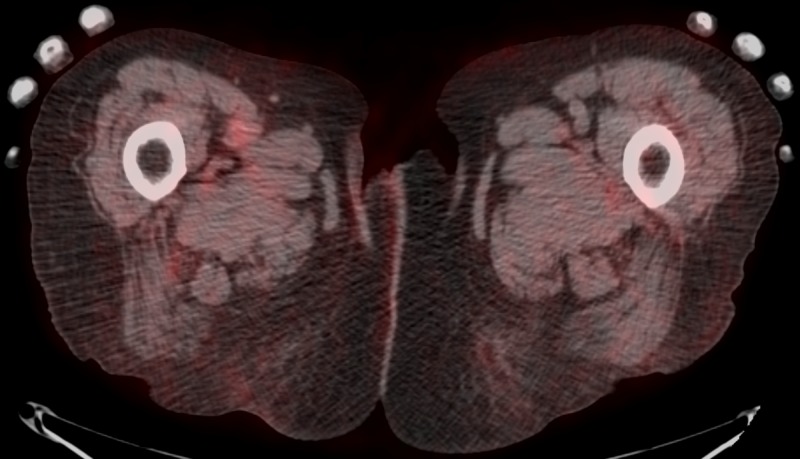
PET/CT December 28, 2015 positron emission tomography-computed tomography: PET/CT

A post-treatment PET/CT of 12/28/15, in comparison to August, now revealed normal PET imaging (Figure 13). An untreated right upper lobe lung lesion originally documented in August was no longer evident, suggestive of an abscopal effect.

**Figure 14:**
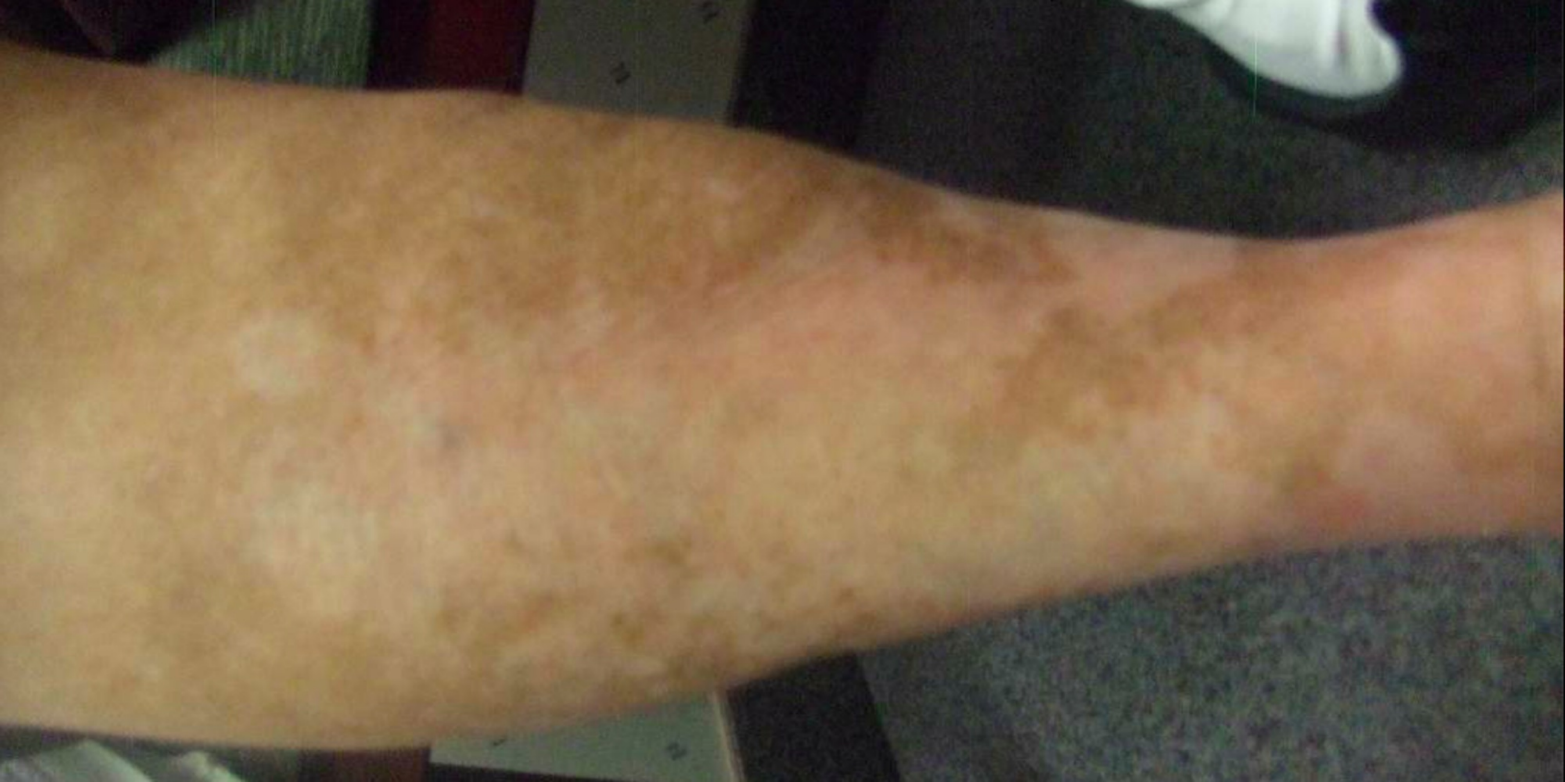
Right leg post treatment

**Figure 15:**
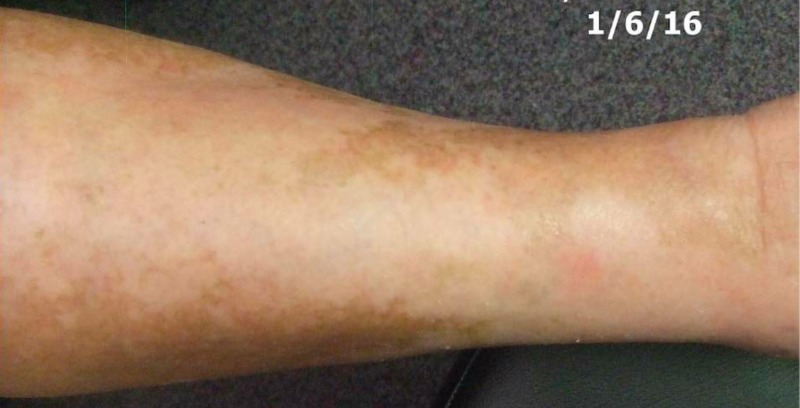
Right leg post treatment

Physical examination in January of 2016 revealed no visible or palpable evidence of the prior malignancy. The right leg returned to normal size and was equal to the left leg (Figures 14-15).

**Figure 16:**
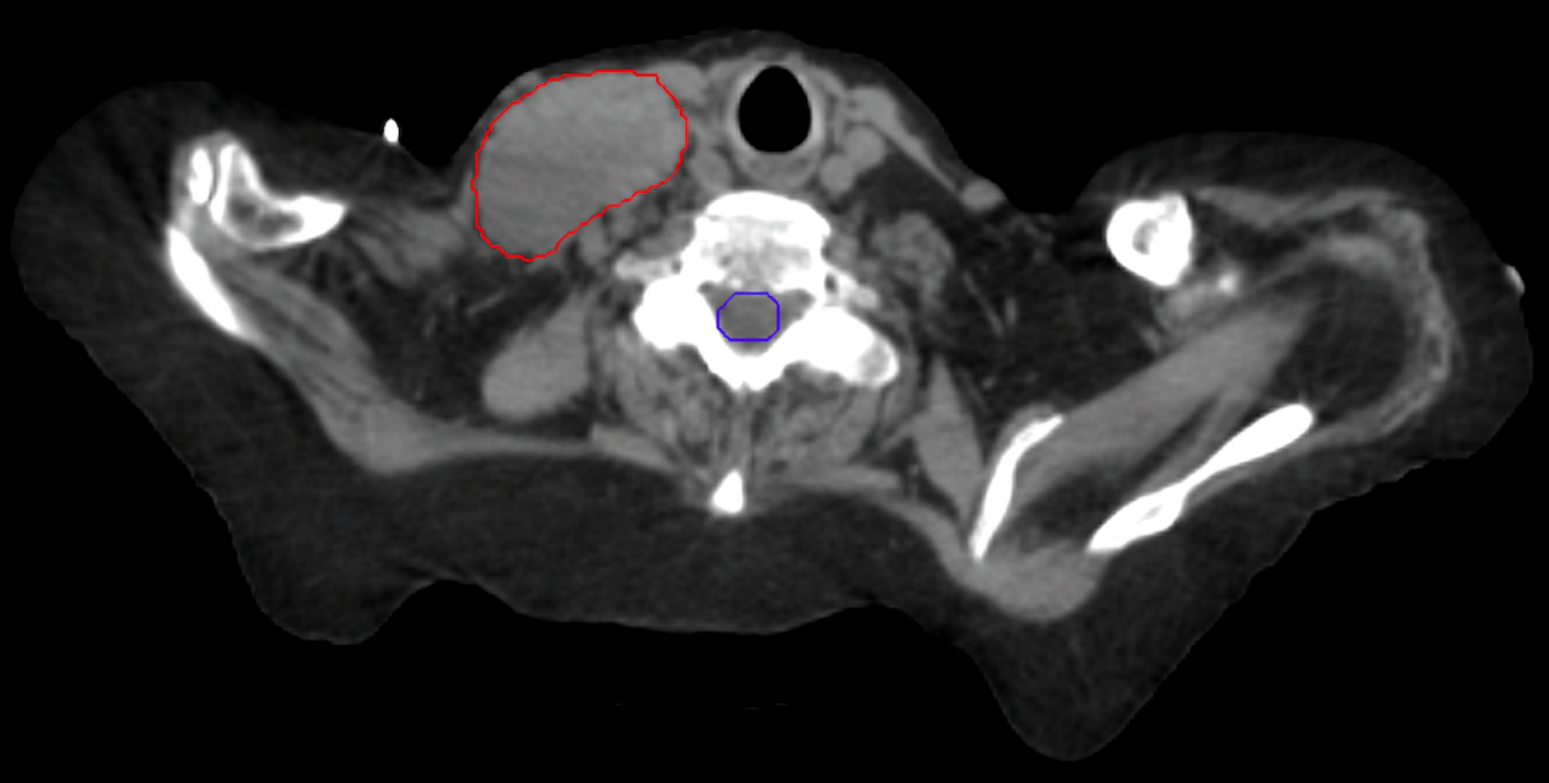
Right neck adenopathy

**Figure 17:**
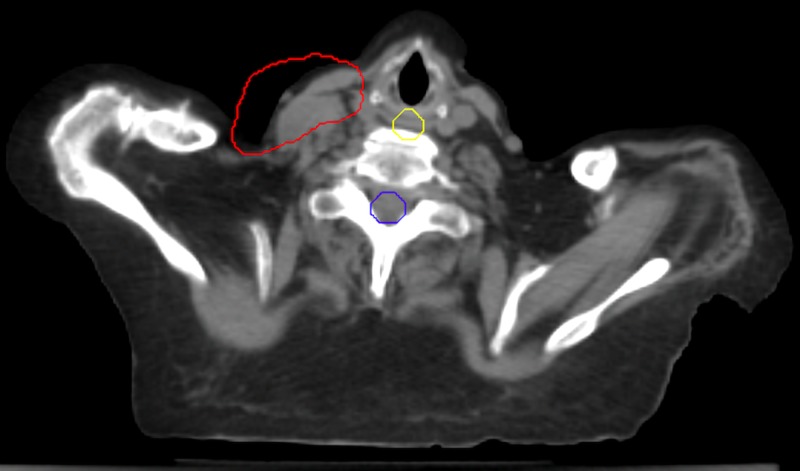
After completion of treatment

However, in latter 2016, the patient was plagued with chronic anemia requiring multiple transfusions. She presented in February of 2017 with episodes of “blacking out.” Physical examination revealed firm right inferior neck adenopathy causing extrinsic compression. This area received only 2250 cGy at 125 cGy per fraction twice daily. In comparison, rapid regression of the neck adenopathy occurred over a two-week period with immediate elimination of vascular compromise (Figures 16-17).

Anemia became profound, as a CT in March revealed diffuse abdominal involvement. Because of her weakened condition, no further treatment was administered, and she passed in April of 2017.

**Figure 18:**
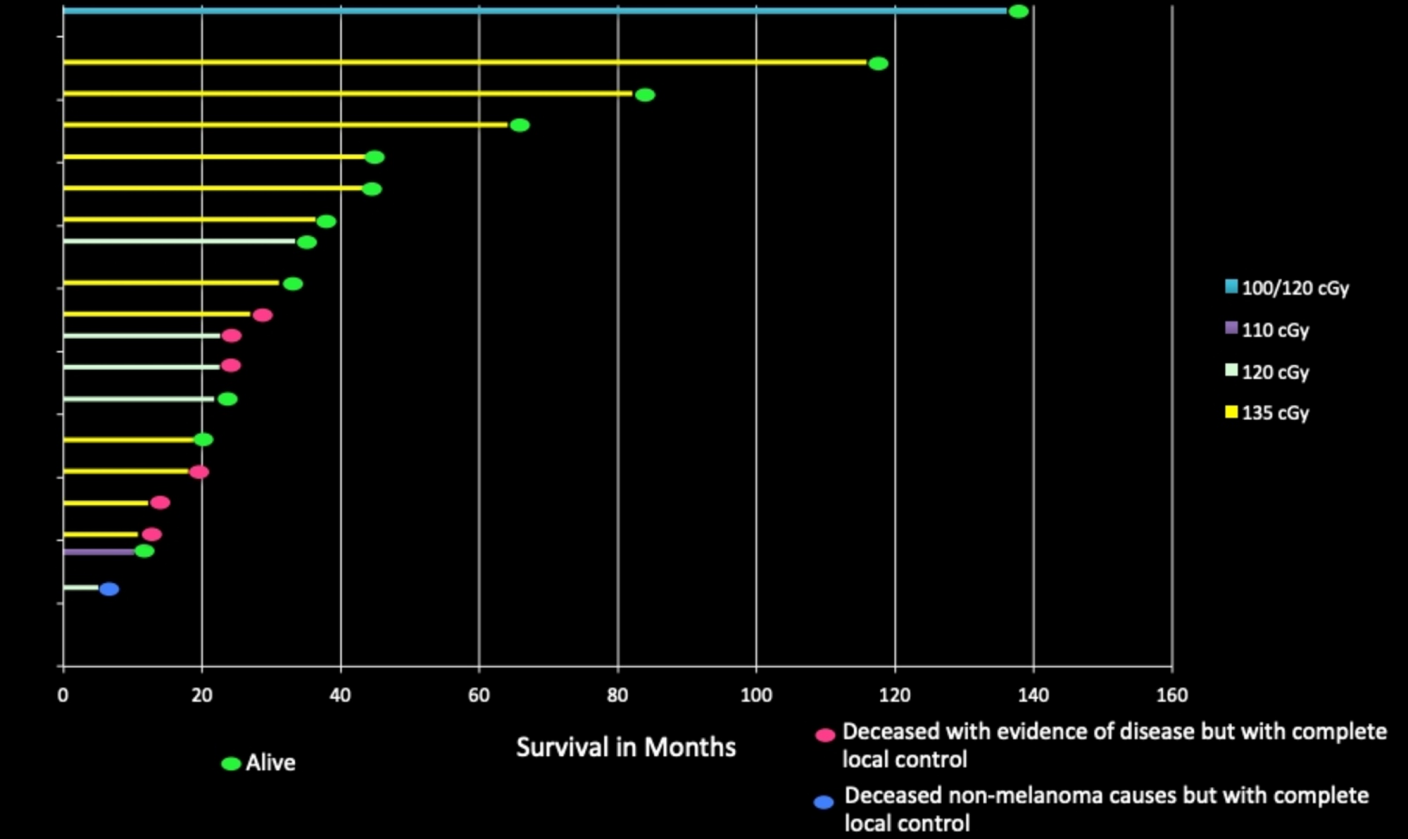
Response/survival from initiation of treatment

The age of patients varied from 45 to 93 at the initiation of treatment. The number of individual sites per person treated was from 1 to 15, with an average of three sites per person; all demonstrating complete control. For every patient treated twice-daily for malignant melanoma, our results indicate a 100% complete response/continued regression and no recurrence within the volume of treatment for locally advanced, visible or radiographic metastatic disease, without the use of systemic intervention (Figure 18). Internal organs partially/completely treated with radiation have included brain, head other than brain, neck, lung, abdomen, liver, pelvis, extremities, as well as multiple areas of adenopathy. Treatment was well tolerated and produced no adverse sequelae.

**Figure 19:**
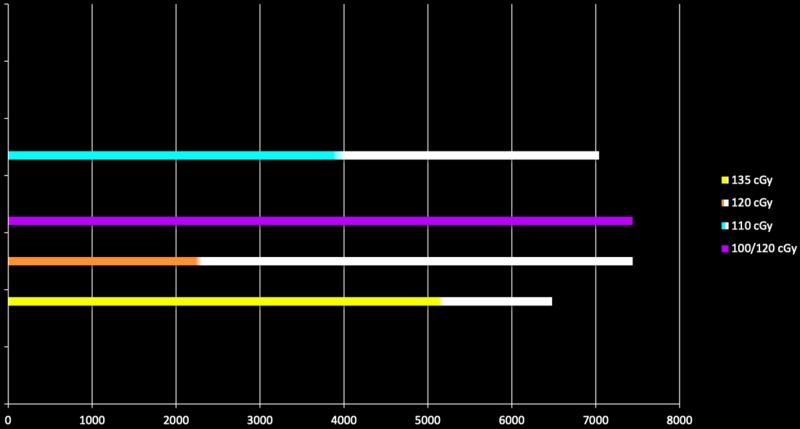
Total dose for each site based on dose per fraction

Figure 19 illustrates the varied range in total dose achieving a complete response for each dose-per-fraction. One would not anticipate that a total dose as low as 3200 cGy would, other than possibly of hematopoietic origin, control local disease for any malignancy. And yet, normal tissue tolerance of specific organs may require such a limited total dose. Knowing that such a low total dose administered twice-daily can achieve control for malignant melanoma now provides the treating physician an expected response not observed with conventional once-daily administration while, at the same time, protect critical organs. Further, non-melanoma malignancies treated twice-daily with similar dose-per-fraction uniformly require higher total doses. That is, a certain total dose must be reached in order to anticipate control, with less total dose increasing malignant viability. This becomes even more divergent for malignant melanoma when its historical response is one of radiation resistance. It is this varied total dose, still achieving local control, that becomes apparent in Figure 19.

## Discussion

For comparison purposes, a total of 62 geographic sites or regions were treated in the studied 19 patients, to include 12 males and 7 females. We define a geographical site or region as a volume of anatomy included within the confines of a single treatment area. That volume may have required different radiation energies and designs based upon ideal physics treatment planning and constraints. The following number of “sites” or “regions” were treated: whole brain (2), head other than brain (5), unilateral neck (6), mediastinum/hilum (3), lung parenchyma/chest wall (10), axilla (3), abdomen/bowel/retroperitoneum (7), liver (3), pelvis (6), and groin/legs/ankles (17). Each separate site may have addressed cutaneous, subcutaneous, muscle, organ, lymphatic, and vascular involvement. Within each region or site, one to six separate, visible, palpable, and/or radiographic lesions were evident.

Those few who fail due to further disease have had involvement in the abdomen. Obviously, the effectiveness of this treatment approach will be influenced, as would immunotherapy/targeted therapy, by the tumor burden at the time of its initiation. However, tumor burdens of 392 cm^3^ in one treated site were completely controlled and as extensive as 700 cm^3^ in individual patients. This represented a group without alternatives for treatment; and yet, their improved survival is significant.

The 21 patients with a subclinical disease were not included for analysis or results. It was the intent of the study to objectively evaluate an obvious response by using a different treatment intervention. Since subclinical disease represents a decreased tumor burden in comparison, such inclusion may have artificially improved the study’s success. However, it is of note that all patients in this group also achieved complete local/regional control and increased survival with radiation alone, exemplifying the positive effect of this approach.

The use of twice-daily treatment for malignant melanoma is a radical change from what is considered “state of the art.” The biologic response seen within these closely defined dose parameters is unanticipated but suggests a potential for cure. It is felt that the greater number of individual treatments may augment a more favorable response, and may correlate with the decreased occurrence of further metastatic disease at a later time. That is, the higher the total treatment delivered using small doses/more fractions, the greater may be the abscopal effect. Thus, this effect may extend beyond what is visibly obvious in the actual study. The nature of malignant melanoma would anticipate a high metastatic rate from isolated localized intervention. The tolerance of normal tissue establishes how much total treatment can be administered. It is, however, this unique combination of the amount administered each time twice-daily, with the increased total number of individual treatments, which may alter this malignancy’s response from what was previously understood. If diffuse metastatic disease is evident at the time of diagnosis, could treating multiple regions increase the abscopal effect by addressing more genetic variants of the original malignancy, now shedding light on the increased survival and potential cure?

Our complete local response from twice-daily radiation could possibly correlate with the abscopal effect. A recent study by Grimaldi et al. reveals a key observation [10]. His report on advanced melanoma showed an abscopal effect in patients treated with immunotherapy (ipilimumab) followed by radiation. Of significant importance, this effect was exclusively observed among patients who displayed a local response to radiation, and 85% of the patients with this local response exhibited an immune-related abscopal effect to radiation. Grimaldi states, "These results suggest RT after ipilimumab may lead to abscopal responses in some patients with advanced melanoma correlating with a prolonged OS. Our data also suggest that local responses to RT may be predictive of abscopal responses." In the present study, although only one patient received immunotherapy prior to twice-daily radiation, all patients achieved a response that was complete. Understanding that Grimaldi's study included immunotherapy as a treatment modality, Liu et al. stated, "a local response to radiation could be of use to prognosticate abscopal effects" [11].

The expense of prior surgeries for any of our patients is unknown. As an example, if Patient One had responded to only an immunotherapeutic agent and lived this time period, which is theoretical for comparison only, radiation would have been delivered at less than 5% of that cost.

Without the use of immune or targeted therapy, our data demonstrate unexpected results after twice-daily radiation. The occurrence of further disease elsewhere over time is markedly diminished, if not eliminated, now approaching and beyond 10 years for some patients. These unforeseen results beg future studies and raise the research challenge of how a uniquely specific immune response may be generated by/for each patient. A remaining question beckons whether immunotherapy, combined with this approach, can further the positive response, progression-free survival, and cure.

## Conclusions

Such an approach of administering radiation treatment twice-daily has never been reported in the medical literature using the above dose range for malignant melanoma. Patients with locally advanced, metastatic palpable, or radiographic visible metastatic malignant melanoma treated twice-daily with radiation therapy using a dose range of 100-135 cGy per fraction achieved a 100% complete response/continued regression with no recurrence within the region of delivery. Of those alive at three years, few demonstrate progression. The results were achieved without systemic intervention, with little, if any side, effects and at a fraction of the alternative’s cost. Evidence of both a visible and an indirect abscopal effect was commonly seen. This observed biologic response is without precedent, and its implications remain to be determined. However, the belief that malignant melanoma is radiation resistant at low doses is now shown to be a false assumption since these cells prove sensitive with twice-daily treatment, harboring an unseen benefit.
